# Associations of therapeutic hypothermia with clinical outcomes in patients receiving ECPR after cardiac arrest: systematic review with meta-analysis

**DOI:** 10.1186/s13049-019-0698-z

**Published:** 2020-01-14

**Authors:** Xi Chen, Zhen Zhen, Jia Na, Qin Wang, Lu Gao, Yue Yuan

**Affiliations:** Department of Cardiology, Beijing Children’s Hospital, Capital Medical University, National Center for Children’s Health, No. 56, Nanlishilu, District Xicheng, Beijing, 100045 China

**Keywords:** Meta-analysis, Therapeutic hypothermia, Cardiac arrest, Neurological outcomes, Survival, Extracorporeal cardiopulmonary resuscitation

## Abstract

**Background:**

Therapeutic hypothermia has been recommended for eligible patients after cardiac arrest (CA) in order to improve outcomes. Up to now, several comparative observational studies have evaluated the combined use of extracorporeal cardiopulmonary resuscitation (ECPR) and therapeutic hypothermia in adult patients with CA. However, the effects of therapeutic hypothermia in adult CA patients receiving ECPR are inconsistent.

**Methods:**

Relevant studies in English databases (PubMed, ISI web of science, OVID, and Embase) were systematically searched up to September 2019. Odds ratios (ORs) from eligible studies were extracted and pooled to summarize the associations of therapeutic hypothermia with favorable neurological outcomes and survival in adult CA patients receiving ECPR.

**Results:**

13 articles were included in the present meta-analysis study. There were nine studies with a total of 806 cases reporting the association of therapeutic hypothermia with neurological outcomes in CA patients receiving ECPR. Pooling analysis suggested that therapeutic hypothermia was significantly associated with favorable neurological outcomes in overall (*N* = 9, OR = 3.507, 95%CI = 2.194–5.607, *P* < 0.001, fixed-effects model) and in all subgroups according to control type, regions, sample size, CA location, ORs obtained methods, follow-up period, and modified Newcastle Ottawa Scale (mNOS) scores. There were nine studies with a total of 806 cases assessing the association of therapeutic hypothermia with survival in CA patients receiving ECPR. After pooling the ORs, therapeutic hypothermia was found to be significantly associated with survival in overall (*N* = 9, OR = 2.540, 95%CI = 1.245–5.180, *P* = 0.010, random-effects model) and in some subgroups. Publication bias was found when evaluating the association of therapeutic hypothermia with neurological outcomes in CA patients receiving ECPR. Additional trim-and-fill analysis estimated four “missing” studies, which adjusted the effect size to 2.800 (95%CI = 1.842–4.526, *P* < 0.001, fixed-effects model) for neurological outcomes.

**Conclusions:**

Therapeutic hypothermia may be associated with favorable neurological outcomes and survival in adult CA patients undergoing ECPR. However, the result should be treated carefully because it is a synthesis of low-level evidence and other limitations exist in present study. It is necessary to perform randomized controlled trials to validate our result before considering the result in clinical practices.

## Background

Cardiac arrest (CA) remains the leading cause of sudden death and is associated with high mortality despite much progress in advanced life support [1–6]. Cardiopulmonary resuscitation using extracorporeal membrane oxygenation (ECPR), also called extracorporeal life support (ECLS), is a modified form of cardiopulmonary bypass. As an alternative resuscitation method, it facilitates the establishment of normal circulation, provides adequate organ perfusion, and then improves both neurological outcomes and survival following CA [7]. Compared with conventional cardiopulmonary resuscitation (CCPR), ECPR is associated with 13% absolute increase of 30 days survival rate in both out-of-hospital cardiac arrest (OHCA) and in-hospital cardiac arrest (IHCA) [7–12]. Technological improvements have made it more accessible and its use has been increased over the past decades, especially in patients with refractory CA [13, 14]. According to the American Heart Association 2015 Guidelines, ECPR may be considered for selected CA patients with potentially reversible etiology [15].

Despite ECPR, the survival rate at hospital discharge with favorable neurological outcomes remains low in CA patients [16–18]. Targeted temperature management (TTM) or mild therapeutic hypothermia via surface cooling has been showed to improve survival and neurological outcomes in patients resuscitated from CA [19, 20]. Cooling the body to 32–34 °C leads to a relative 35% increase in survival compared with no intervention [21]. International guidelines strongly recommend initiating therapeutic hypothermia for eligible CA patients to improve outcomes [22–24]. ECPR is an ideal tool for rapid and homogenous cooling and can also be augmented with surface cooling. Up to now, there are some studies evaluating the combined use of ECPR and therapeutic hypothermia in adult CA patients and/or comparing therapeutic hypothermia treatment with no therapeutic hypothermia induction [9, 25–37]. However, the sample size of those studies is limited, the proportion of patients receiving therapeutic hypothermia ranges variously, and no conclusive result is derived whether there is a benefit of therapeutic hypothermia treatment in CA patients undergoing ECPR.

In the present study, for the first time, we performed a systematic review with meta-analysis to compare neurological outcomes and survival (Outcomes) between therapeutic hypothermia treatment at 32–34 °C (Intervention) and any other temperature controls including no therapeutic hypothermia induction and alternative targeted temperatures range (> 34 °C, ≤36 °C) (Control) in adult CA patients receiving ECPR (Population).

## Materials and methods

The results of this meta-analysis were written according to the PRISMA (Preferred Reporting Items for Systematic Reviews and Meta-Analysis) reporting guidelines for randomized trials and MOOSE (Meta-analysis Of Observational Studies in Epidemiology) reporting guidelines for observational studies. We used the PICO (Participants/Population, Intervention/Exposure, Comparison/Control, and Outcomes) scheme to generate the research questions, create a search strategy, and guide study selection. Participants/Population, adult CA patients undergoing ECPR; no restrictions were applied to downtime, bystander CPR attempt, or witnessed arrest. Intervention/Exposure, therapeutic hypothermia with a targeted temperature of 32–34 °C. Comparison/Control, any temperature controls (> 34 °C) including no therapeutic hypothermia induction and alternative targeted temperatures range (> 34 °C, ≤36 °C). Outcomes, [1] favorable neurological outcome at hospital discharge/30 days/90 days, which were evaluated by the cerebral performance category (CPC) with CPC1–2 as favorable outcomes [2]; survival at hospital discharge/30 days/90 days.

### Publication search

A systematic search of relevant articles on the associations of therapeutic hypothermia with clinical outcomes (survival or neurological outcomes) in CA patients undergoing ECPR published from January 2000 to September 2019 was carried out in four English databases (PubMed, OVID, Embase, and ISI Web of Science). The following searching keywords were used, “extracorporeal membrane oxygenation OR extracorporeal oxygenation OR extracorporeal circulation OR extracorporeal cardiopulmonary resuscitation OR extracorporeal life support OR ECMO OR ECPR OR E-CPR OR ECLS”, “out-of-hospital cardiac arrest OR in-hospital cardiac arrest OR cardiac arrest OR heart arrest OR OHCA OR IHCA”, and “Hypothermia OR therapeutic hypothermia OR induced hypothermia OR mild hypothermia OR cooling OR cryotherapy OR targeted temperature management OR TTM”. The retrieved articles were screened and selected according to the inclusion and exclusion criteria by two independent investigators. The references of the selected articles and the review articles were also screened to identify additional eligible studies.

### Inclusion and exclusion criteria

Inclusion criteria, [1] subjects were adult patients with CA undergoing ECPR [2]; the clinical outcomes reported in the studies were neurological outcomes (primary outcomes) and patients’ survival (secondary outcomes) at hospital discharge/30 days/90 days; the neurological outcomes should be assessed by Cerebral Performance Categories (CPC) score and CPC 1 or 2 was defined as favorable outcomes while CPC 3–5 were defined as poor outcomes [38] [3]; the target temperature of therapeutic hypothermia was stated [4]; the clinical outcomes between therapeutic hypothermia intervention and controls were compared [5]; unadjusted or adjusted odds ratios (ORs) with their 95% confidence intervals reflecting the associations of therapeutic hypothermia with clinical outcomes were reported or the proportions of the patients with unfavorable and favorable outcomes in therapeutic hypothermia and control arms were reported [6]; the language was limited as English. Exclusion criteria, [1] studies included other disease besides CA and the results for CA could not be separated [2]; studies performed in neonates/children/pediatric patients [3]; studies included other treatments besides ECPR (such as CCPR) and results for ECPR could not be separated [4]; studies with insufficient data [5]; studies with sample size< 10 [6]; studies with duplicate data; the author information, study centers, population, and the results of the candidate studies were compared to identify the studies with fully or partly duplicate data; when studies with duplicate data were identified, the studies with larger sample size and more recently published were included [7]; all patients were treated with therapeutic hypothermia (i.e. no controls) [8]; the target temperature of therapeutic hypothermia was not reported [9]; not original full articles, such as abstracts, letters, or review articles; and [10] case series or case report.

### Data extraction

The following information was collected by two authors independently at the same time from each eligible study, the author information, publication year, countries, study period, gender, age, sample size, location of CA, bystander CPR attempt, rhythm, follow-up points, outcomes, indication criteria of ECPR and therapeutic hypothermia, target temperature of therapeutic hypothermia and control arms, the comparability of baseline characteristics, CA characteristics and neurological status before therapeutic hypothermia treatment, patients number with unfavorable/favorable outcomes in therapeutic hypothermia and control arms or direct ORs with 95%CIs, and analysis method (univariate or multivariate analysis). We also tried to contact with the authors of the included studies for detailed results needed in our study. Disagreement was solved by discussion and if the disagreement was still not solved, the arbitration would be performed by a third author.

### Quality score assessment

The methodological quality of each included article was evaluated by a modified Newcastle Ottawa Scale (mNOS) with scores ranging from 0 to 9 (Additional file 1: Table S1). mNOS score ≥ 7 was considered as high quality.

### Statistical analysis

The odds ratios (ORs) with 95% CIs from each eligible study were pooled to assess the strength of the associations of therapeutic hypothermia with neurological outcomes or survival in adult CA patients undergoing ECPR. If 30 days endpoints were not reported, discharge endpoints were used. The longest available follow-up endpoints were used in overall analysis and all follow-up endpoints were used when performing subgroup analysis according to follow-up period. The association was considered significant if *P* < 0.05 by Z-test. Chi-square based on Q statistic test was used to quantify the heterogeneity in each analysis. If P>0.1/I^2^<50%, ORs were pooled with fixed-effects model (Mantel-Haenszel method); otherwise, the random-effects model (DerSimonian-Laird method) was used [39]. Begg’s funnel plots and Egger’s test were used to determine the potential publication bias [40, 41]. The Duval and Tweedie’s trim-and-fill method, a funnel plot-derived and two-step method, was used to estimate the effect size accounting for publication bias. The stability of the pooling result was verified by sensitivity analysis through omitting each study in each turn. In addition, subgroup analyses according to control type, regions, sample size, CA location, ORs obtained methods, follow-up period, and mNOS scores were also performed. Stata 12.0 software (Stata Corp, College Station, TX, USA) was used to perform all the analyses.

## Results

### Identification of studies

**Fig. 1:**
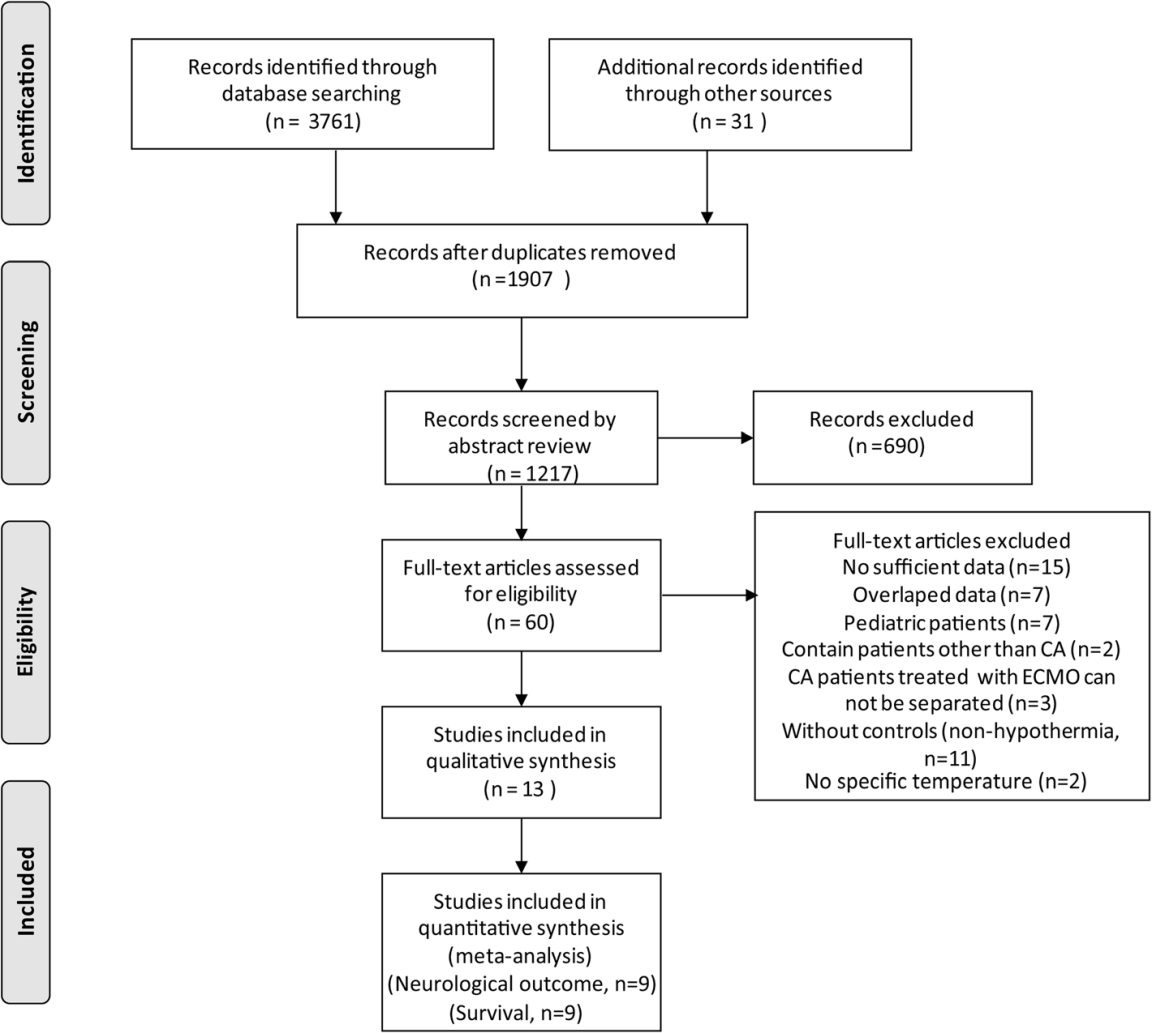
Flow chart of study selection

Figure 1 showed the literature searching and screening process. According to the searching strategy, a total of 3792 documents were initially retrieved from four English databases (PubMed, OVID, Embase and ISI Web of Science) and additional sources. After excluding the duplicated papers, review or meeting abstracts, and irrelevant articles, 60 articles were left for further screening by checking the full texts. 47 articles were excluded, of which, seven were overlapped studies [42–48], seven were performed in pediatric patients [49–55], two study contained patients with other disease besides CA and the data related to CA could not be obtained separately [16, 56], three studies contained CA patients treated with other methods besides ECPR and the data related to ECPR could not be obtained separately [17, 57, 58], fifteen studies had no sufficient data [59–73], and eleven studies only had therapeutic hypothermia arms (without controls) [37, 74–83]. Two studies did not state the specific temperature of therapeutic hypothermia and control arms [84, 85]. Finally, 13 papers containing 1159 cases were included in the present study [9, 25–36]. Notably, two papers published by Kagawa et al. in 2012 and 2015 with some overlapped participants were both included because they reported different clinical outcomes [25, 27]. Studies by Kim et al. in 2014 [26] and Han et al. in 2019 [35] also had overlapped participants, however, the article in 2019 only reported patients’ survival, thus the article in 2014 was included when analyzing neurological outcomes. Goto et al. [33] and Otani et al. [34] performed studies on partly overlapped population. Goto et al. collected the patients from 2005 to 2013 and reported both neurological and survival outcomes while Otani et al. collected the patients from 2009 to 2017 and reported only neurological outcomes. Thus, the study by Goto et al. was included when analyzing patients’ survival whereas the study by Otani et al. was included when analyzing patients’ neurological outcomes according to inclusion and exclusion criteria.

### Study characteristics

The detailed characteristics of the included 13 articles were shown in Table 1 and Additional file 2: Table S2. The studies were published from 2012 to 2019 and the samples size ranged from 10 to 274. The studies were conducted in Korea (*n* = 5), Japan (*n* = 6), Australia (*n* = 1), and Singapore (*n* = 1). The proportion of patients receiving therapeutic hypothermia, with favorable neurological outcomes, and with survival ranged from 13.14 to 78.26%, 13.92 to 35.14%, and 14.00 to 43.48%, respectively. Seven studies enrolled only OHCA patients and six studies enrolled both IHCA and OHCA patients. Nine studies reported the association of therapeutic hypothermia with neurological outcomes in adults CA patients undergoing ECPR [9, 26–28, 30–32, 34, 36], and nine reported the association of therapeutic hypothermia with patients’ survival [9, 25, 28–31, 33, 35, 36]. Quality assessment was performed according to a modified NOS (Additional file 1: Table S1). The mNOS scores ranged from 6 to 9 for neurological outcomes (Additional file 1: Table S3) and from 6 to 8 for survival outcomes (Additional file 1: Table S4).

**Table 1:** Baseline characteristics of the included studies

Author	Region	Period	Gender (F/M)	Age (yrs.)	IHCA	OHCA	Witnessed CA	Bystander CPR attempt	Shockable rhythm	TH cases	TH temperature	Control temperature	Neurological outcomes	Survival	Follow-up
Kagawa et al., 2012	Japan	2004–2011	16/70	63.0	44	42	77	67	46	32	34 °C	> 34 °C	NO	YES	30 days
Maekawa et al., 2013	Japan	2000–2004	9/44	54.5	0	53	53	29	32	25	34 °C	> 34 °C	YES	YES	3 months
Kim et al., 2014	Korea	2006–2013	12/40	53.0	0	52	42	22	31	14	33 °C	Not induced	YES	NO	3 months
Kagawa et al., 2015	Japan	2003–2014	23/64	61.8	33	54	82	66	40	48	< 34 °C	> 34 °C	YES	NO	3 months
Choi et al., 2016	Korea	2011–2015	3//7	57.2	0	10	10	8	3	6	33 °C	Not induced	YES	YES	30 days
Lee et al., 2016	Korea	2009–2014	3//20	55.0	0	23	23	14	10	18	33–34 °C	Not induced	NO	YES	30 days
Dennis et al., 2017	Australia	2009–2016	10/27	54.0	25	12	27	30	19	15	33 °C	36 °C	YES	YES	Hospital discharge
Pang et al., 2017	Singapore	2003–2016	17/62	49.9	73	6	73	NA	33	14	34 °C	Not induced	YES	YES	Hospital discharge
Yukawa et al., 2017	Japan	2011–2015	14/65	59.0	0	79	79	46	58	50	34 °C	Not induced	YES	NO	Hospital discharge
Goto et al., 2018	Japan	2005–2013	25/119	63.0	0	144	25	54	88	63	34 °C	> 34 °C	NO	YES	30 days
Otani et al., 2018	Japan	2009–2017	20/115	65.0	0	135	135	74	87	28	34 °C	36 °C	YES	NO	Hospital discharge
Han et al., 2019	Korea	2006–2017	26/74	55.5	25	75	86	73	54	26	33 °C	Not induced	NO	YES	Hospital discharge
Ryu et al., 2019	Korea	2004–2016	100/174	62.0	250	24	250	272	143	36	32–34 °C	> 34 °C	YES	YES	Hospital discharge

Abbreviations: *CA* cardiac arrest; *IHCA* in-hospital cardiac arrest; *OHCA* out-of-hospital cardiac arrest; *CPR* cardiopulmonary resuscitation; *TH* therapeutic hypothermia; *F* female; *M* male; *NA* not applicable

### Meta-analysis results

#### Association of therapeutic hypothermia with neurological outcomes in CA patients receiving ECPR

**Fig. 2:**
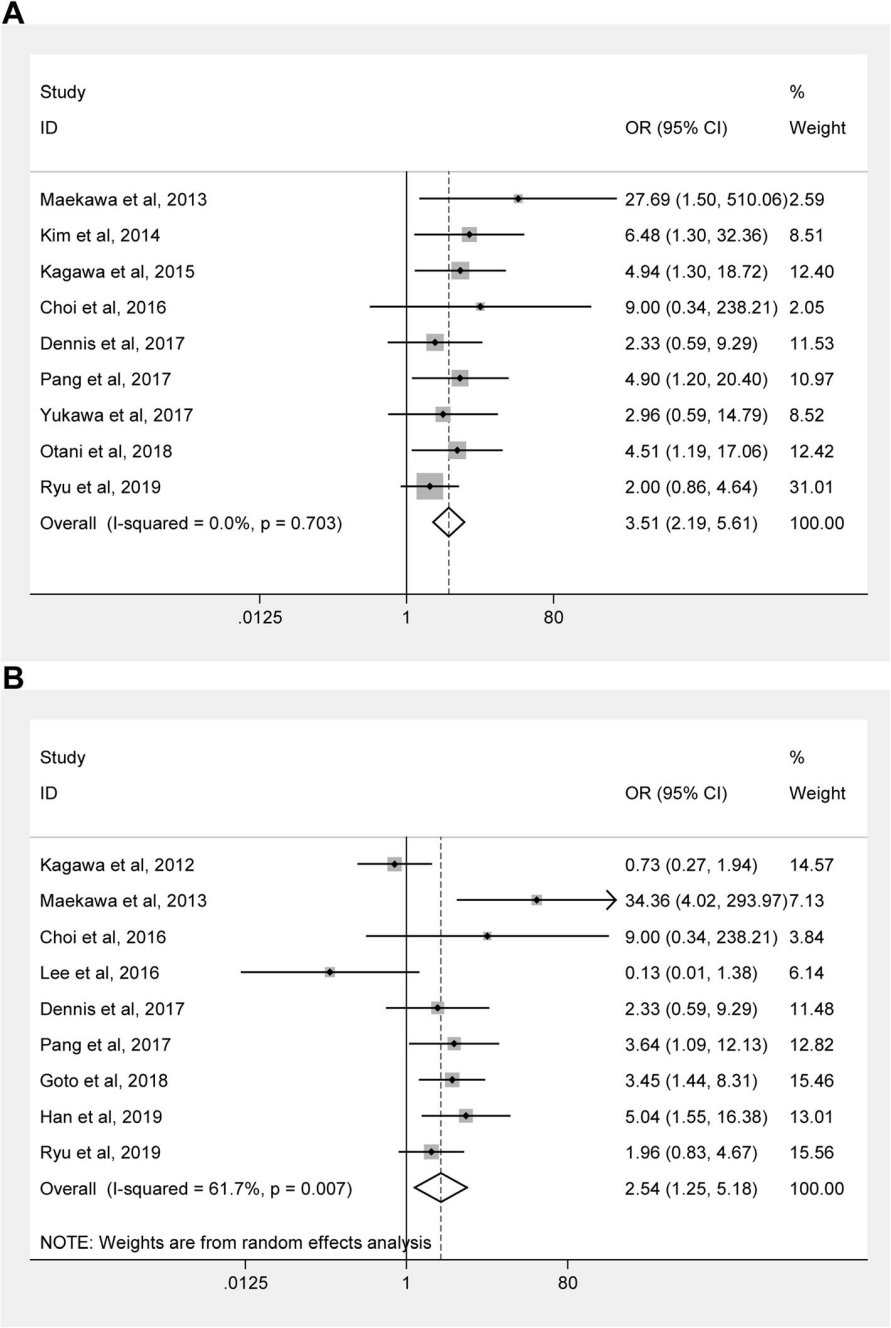
Forest plots for the associations of therapeutic hypothermia with favorable neurological outcomes (**a**) and survival (**b**) in adult cardiac arrest patients undergoing ECPR

There were nine studies with a total of 806 cases reporting the association of therapeutic hypothermia with neurological outcomes in CA patients receiving ECPR. Six studies reporting neurological outcomes at hospital discharge and three studies reporting that at 90 days. Pooling analysis suggested that therapeutic hypothermia was significantly associated with favorable neurological outcomes in CA patients receiving ECPR in overall (*N* = 9, OR = 3.507, 95%CI = 2.194–5.607, *P* < 0.001, fixed-effects model) and in all subgroups according to control type, regions, sample size, CA location, ORs obtained methods, follow-up period, and mNOS scores (Fig. 2a and Table 2).

**Table 2:** Association of therapeutic hypothermia with favorable neurological outcomes in CA patients receiving ECPR

Subgroup	N	OR	LCI	UCI	P_OR_	Model	P_hetero_	I^2^	P_begg_	P_egger_
Overall	9	3.507	2.194	5.607	< 0.001	F	0.703	0.00%	0.048	0.008
Control type										
Other temperature controls	5	3.067	1.750	5.374	< 0.001	F	0.387	3.50%		
Non-TH induction	4	4.794	2.037	11.282	< 0.001	F	0.891	0.00%		
Regions	1									
Japan	4	4.803	2.196	10.505	< 0.001	F	0.627	0.00%		
Korea	3	2.740	1.324	5.673	0.007	F	0.342	6.70%		
Sample size	1									
< 80	4	4.814	1.873	12.378	0.001	F	0.438	0.00%		
≥ 80	5	3.162	1.841	5.428	< 0.001	F	0.697	0.00%		
CA location										
Mixed	4	2.828	1.587	5.039	< 0.001	F	0.577	0.00%		
OHCA	5	5.320	2.382	11.881	< 0.001	F	0.744	0.00%		
mNOS score	1									
≥ 7	5	3.456	1.968	6.070	< 0.001	F	0.374	5.70%		
< 7	4	3.627	1.553	8.468	0.003	F	0.741	0.00%		
OR obtained methods										
U	6	4.428	2.211	8.870	< 0.001	F	0.699	0.00%		
M	3	2.885	1.527	5.449	0.001	F	0.428	0.00%		
Follow-up	1									
Discharge/30 days	6	2.889	1.690	4.940	< 0.001	F	0.813	0.00%		
90 days	3	6.592	2.505	17.349	< 0.001	F	0.573	0.00%		

Abbreviations: *TH* therapeutic hypothermia; *CA* cardiac arrest; *IHCA* in of hospital cardiac arrest; *OR* odds ratios; 95%*CI* 95% confidence interval; *LCI* lower 95% confidence interval; *UCI* upper 95% confidence interval; *F* fixed-effects model; *R* random-effects model; P_hetero_, *P*-value for heterogeneity; P_begg_, *P*-value for Begg’s test; P_egger_, *P*-value for Egger’s test; *U* univariate analysis; *M* multivariate analysis; *mNOS* modified Newcastle-Ottawa scale

#### Association of therapeutic hypothermia with survival in CA patients receiving ECPR

There were nine studies with a total of 806 cases assessing the association of therapeutic hypothermia with survival in CA patients receiving ECPR. After pooling the ORs, therapeutic hypothermia was found to be significantly associated with favorable survival in overall (*N* = 9, OR = 2.540, 95%CI = 1.245–5.180, *P* = 0.010, random-effects model; Fig. 2b and Table 3). After subgroup analysis according to control type, regions, sample size, CA location, ORs obtained methods, follow-up period, and mNOS scores, significant associations were found in subgroups of other temperature control (*N* = 5, *P* = 0.039, random-effects model), larger sample size (*N* = 5, P = 0.010, random-effects model), mixed CA location (*N* = 4, *P* = 0.048, fixed-effects model), OHCA (*N* = 5, *P* = 0.050, random-effects model), univariate analysis obtained ORs (*N* = 8, *P* = 0.025, random-effects model), 30 days/at discharge survival (*N* = 8, *P* = 0.023, random-effects model), and high mNOS scores (*N* = 4, *P* < 0.001, fixed-effects model).

**Table 3:** Association of therapeutic hypothermia with survival in CA patients receiving ECPR

Subgroup	N	OR	LCI	UCI	P_OR_	Model	P_hetero_	I^2^	P_begg_	P_egger_
Overall	9	2.540	1.245	5.180	0.010	R	0.007	61.70%	0.754	0.703
Control type										
Other temperature controls	5	2.542	1.046	6.174	0.039	R	0.016	67.40%		
Non-TH induction	4	2.364	0.564	9.919	0.240	R	0.046	62.50%		
Regions										
Japan	3	3.478	0.625	19.373	0.155	R	0.002	83.60%		
Korea	4	1.953	0.522	7.308	0.320	R	0.043	63.20%		
Sample size										
< 80	4	2.984	0.328	27.124	0.332	R	0.007	75.50%		
≥ 80	5	2.370	1.230	4.567	0.010	R	0.075	52.90%		
CA location										
Mixed	4	1.703	1.004	2.889	0.048	F	0.193	36.50%		
OHCA	5	3.759	1.001	14.122	0.050	R	0.015	67.60%		
mNOS score										
≥ 7	4	3.248	1.909	5.525	< 0.001	F	0.112	49.90%		
< 7	5	1.540	0.461	5.141	0.482	R	0.019	66.10%		
OR obtained methods										
U	8	2.683	1.134	6.347	0.025	R	0.004	66.10%		
M	1									
Follow-up										
Discharge/30 days	8	2.106	1.106	4.008	0.023	R	0.040	52.40%		
90 days	1									

Abbreviations: *TH* therapeutic hypothermia; *CA* cardiac arrest; *IHCA* in of hospital cardiac arrest; *OR* odds ratios; 95%*CI* 95% confidence interval; *LCI* lower 95% confidence interval; *UCI* upper 95% confidence interval; *F* fixed-effects model; *R* random-effects model; P_hetero_, *P*-value for heterogeneity; P_begg_, *P*-value for Begg’s test; P_egger_, *P*-value for Egger’s test; *U* univariate analysis; *M* multivariate analysis; *mNOS* modified Newcastle-Ottawa scale

### Heterogeneity, sensitivity analysis and publication bias

**Fig. 3:**
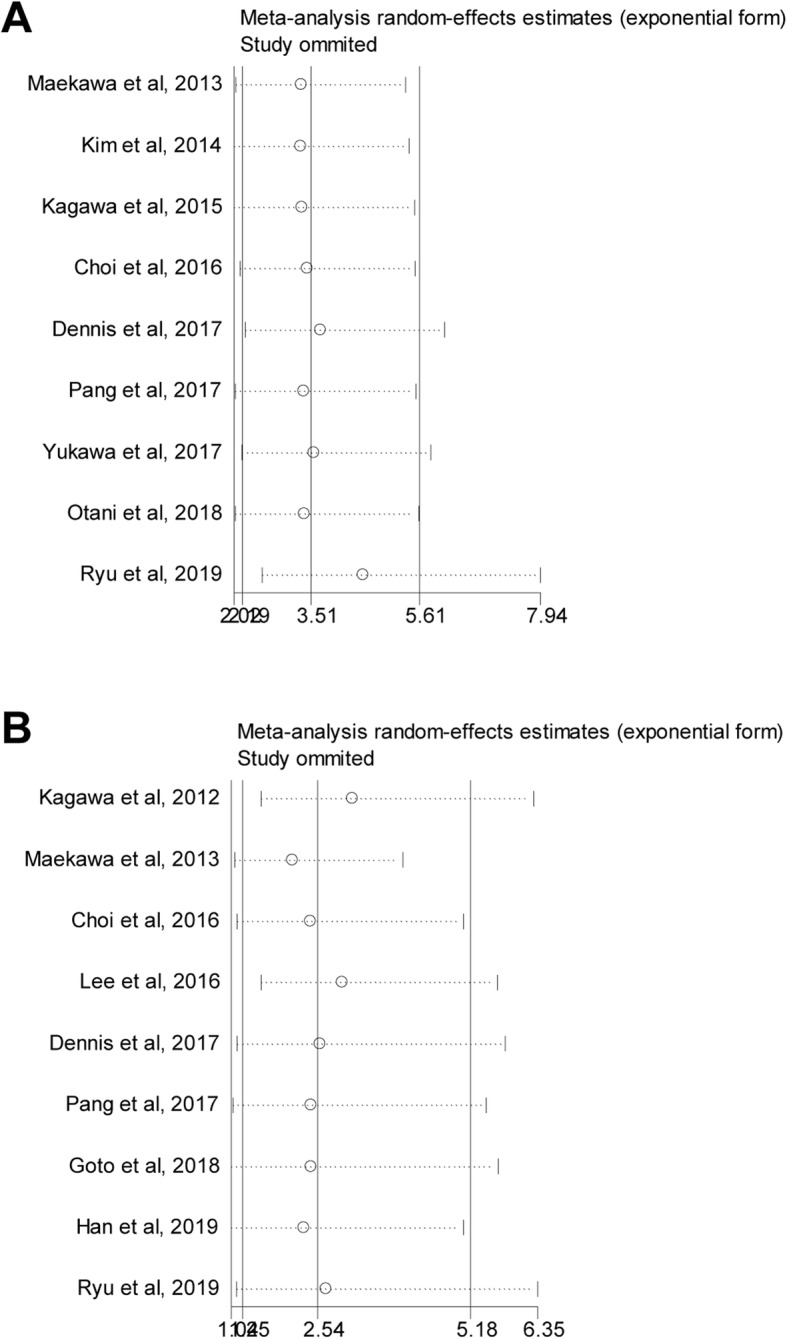
Sensitivity analysis of the associations of therapeutic hypothermia with favorable neurological outcomes (**a**) and survival (**b**) in adult cardiac arrest patients undergoing ECPR

**Fig. 4:**
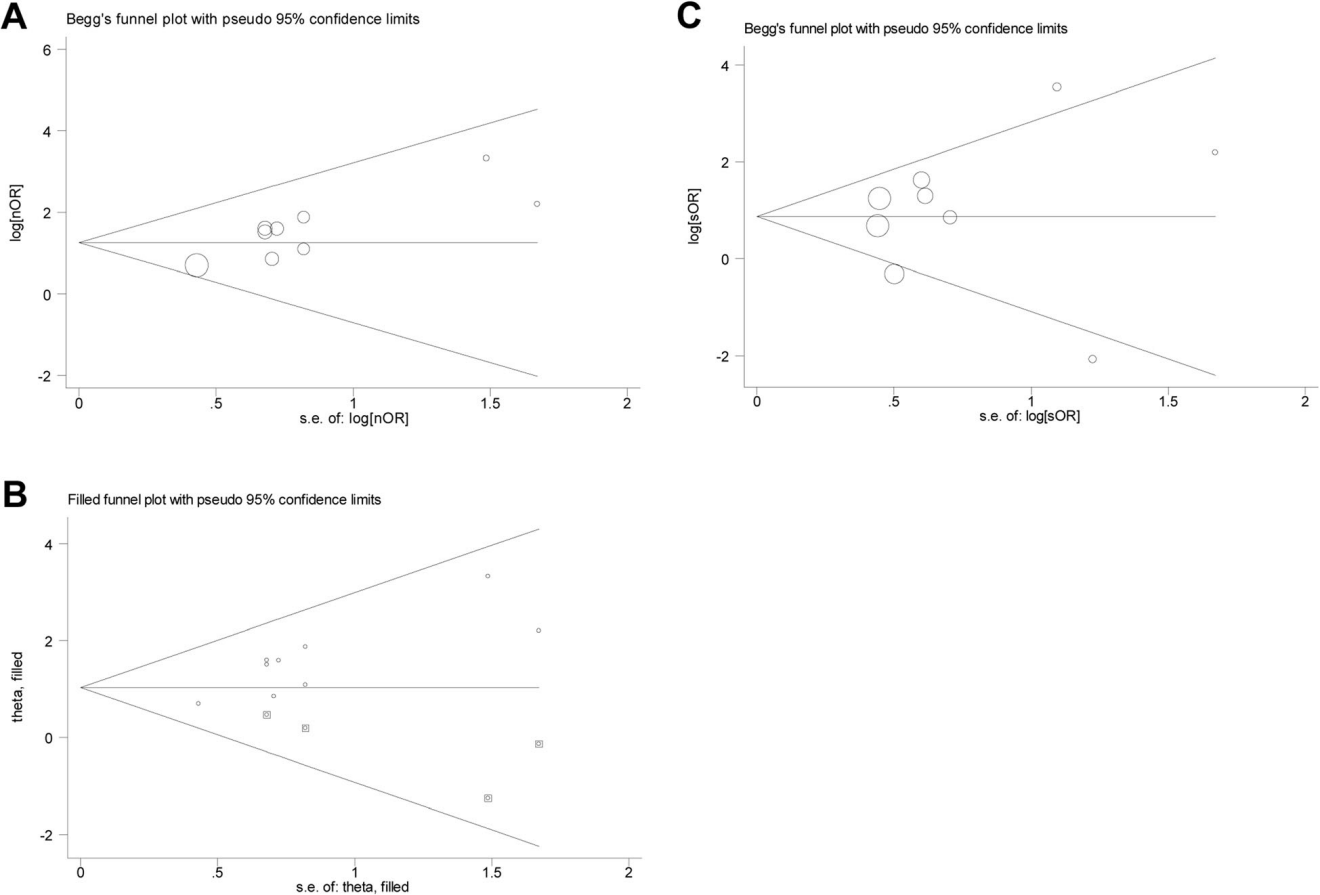
Begg’s funnel plots for publication bias analysis. **a** Funnel plot for the association of therapeutic hypothermia with favorable neurological outcomes in adult cardiac arrest patients undergoing ECPR. **b** Trim-and-fill analysis for the association of therapeutic hypothermia with favorable neurological outcomes. **c** Funnel plot for the association of therapeutic hypothermia with survival in adult cardiac arrest patients undergoing ECPR

No heterogeneity existed when assessing the association of therapeutic hypothermia with neurological outcome while heterogeneity was found when evaluating the association of therapeutic hypothermia with patients’ survival. After deleting the studies by Kagawa et al. in 2012 and Lee et al. in 2016, the heterogeneity reduced to insignificance and the association of therapeutic hypothermia with patients’ survival was still significant (*N* = 7, OR = 3.410, 95%CI = 2.168–5.362, *P* < 0.001, fixed-effects model). Sensitivity analysis was performed to examine the influence of individual study on the pooling ORs and revealed almost the same results (Fig. 3a and b). Begg’s and Egger’s tests were used to assess the publication bias. Publication bias was found when evaluating the association of therapeutic hypothermia with neurological outcomes (Fig. 4a and Table 2; P_begg_ = 0.048, P_egger_ = 0.008). Trim-and-fill analysis suggested that there were four “missing” studies on the bottom side of the funnel plot (Fig. 4b). After adjustment, therapeutic hypothermia was still significantly associated with favorable neurological outcomes in CA patients receiving ECPR (OR = 2.800, 95%CI = 1.842–4.526, *P* < 0.001, fixed-effects model). Conversely, no publication bias was found when evaluating the association of therapeutic hypothermia with patients’ survival (Fig. 4c and Table 3; P_begg_ = 0.754, P_egger_ = 0.703).

## Discussion

Therapeutic hypothermia can decrease cerebral metabolism, alleviate the ischemic loss of ion gradients, reduce free radicals, and decrease postischemic inflammation after cerebral ischemia, thus protect patients against hypoxic brain damage following CA [86, 87]. Therapeutic hypothermia has been recommended for eligible CA patients in order to improve outcomes and is increasingly used [22–24]. However, whether it improves neurological outcomes and survival in ECPR treated CA patients is not conclusive and the results in recent research are not consistent. In addition, therapeutic hypothermia can bring significant major complications including coagulation dysfunction, infection, and other systemic problems [88]. This promoted us, for the first time, to perform a systematic review with meta-analysis to summarize the effects of therapeutic hypothermia in CA patients receiving ECPR, and we found therapeutic hypothermia might be associated with favorable neurological outcomes and survival in CA patients receiving ECPR in overall.

Therapeutic hypothermia is intended to be performed in CA patients with poor neurological status. Of the 13 included studies, seven studies describe the indications of therapeutic hypothermia treatments [9, 25, 27, 29, 31, 34, 36]. All the unconscious patients after ECPR are recommended receiving therapeutic hypothermia in six studies and one study [31] randomly divides the unconscious CA patients into therapeutic hypothermia group and non-therapeutic hypothermia group. In other words, the patients in therapeutic hypothermia arms usually have a poorer neurological status at baseline than those in control arms, which might act as a bias when evaluating the associations of therapeutic hypothermia with patients’ outcomes. We pooled the seven studies and found that therapeutic hypothermia was associated with patients’ favorable neurological outcomes (*N* = 4, OR = 3.627, 95%CI = 1.533–8.468, *P* = 0.003, fixed-effects model) and survival (*N* = 4, OR = 3.248, 95%CI = 1.909–5.525, *P* < 0.001, fixed-effects model), indicating that although the patients in therapeutic hypothermia arm might have a poorer baseline neurological status, an improved prognosis was still identified.

In 2002, two randomized clinical trials (RCTs) suggest that therapeutic hypothermia at 33 °C improves patients’ neurological outcomes compared to any temperature controls in CA [19, 89]. Recently, Nielsen et al. perform a large RCT and find no differences of patients’ neurological outcomes and survival between target temperature managements at 33 °C and 36 °C [90]. However, it is difficult to extrapolate this result to the ECPR population. In 2015, Kagawa et al. perform an observational study on 237 patients receiving targeted temperature management after CA and find no difference of neurological outcomes between targeted temperatures management at < 34 °C and at ≥34 °C in entire CA patients [27]. Nevertheless, they find < 34 °C is associated with improved neurological outcomes in combined ECPR treated CA patients. In the present study, we observed improved neurological outcomes after therapeutic hypothermia induction at ≤34 °C compared to no therapeutic hypothermia induction (*N* = 4, OR = 4.794, 95%CI = 2.037–11.28, *P* < 0.001, fixed-effects model) or alternative targeted temperature controls (> 34 °C) (*N* = 5, OR = 3.067, 95%CI = 1.750–5.374, *P* < 0.001, fixed-effects model), respectively, in CA patients undergoing ECPR.

Therapeutic hypothermia is associated with OHCA patients compared with IHCA patients in practice [61]. In the 13 included studies, the proportion of patients receiving therapeutic hypothermia ranges from 20.74 to 78.26% with an average of 41.13% in studies enrolling only OHCA patients and from 13.14 to 55.17% with an average of 25.79% in studies enrolling both IHCA and OHCA patients. And in patients receiving therapeutic hypothermia, the proportion of those with favorable neurological outcomes ranges from 13.92 to 30.00% with an average of 15.80% in studies enrolling only OHCA patients and from 19.54 to 35.13% with an average of 26.00% in studies enrolling both IHCA and OHCA patients. Similar results are also observed in the studies in which all CA patients received therapeutic hypothermia and ECPR [30, 37, 74–82]. As OHCA is associated with worse outcomes compared to IHCA [45, 62, 91], the proportion of OHCA patients in studies enrolling both OHCA and IHCA patients may influence the analysis of the associations of therapeutic hypothermia with neurological outcomes and survival in CA patients undergoing ECPR. The pooling ORs for neurological outcomes in subgroup enrolling only OHCA and subgroup enrolling both OHCA and IHCA are 5.320 and 2.828, respectively. The pooling ORs for patients’ survival in subgroup enrolling only OHCA and subgroup enrolling both OHCA and IHCA are 3.759 and 1.703, respectively.

Selection criteria for starting ECPR and characteristics of CA including cause of CA may also affect our results stability. Almost of our enrolled studies have stated the ECPR inclusion and exclusion criteria (Additional file 2: Table S2), however, these criteria are diverse, and we can’t solve this problem based on current data. Characteristic of CA, such as the proportion of the patients with IHCA, witnessed CA, bystander CPR attempt, shockable rhythm, cardiac origin CA, or acute coronary syndrome, are also different in our enrolled studies (Additional file 2: Table S2). We have used a meta-regression method to explore the effects of the characteristic of CA on the association of therapeutic hypothermia with neurological outcomes in CA patients receiving ECPR and find these characteristics have no effect on their association (Additional file 1: Table S5).

There are other shortcomings that may impact our results stability. Firstly, the eligible studies and sample size are relative limited although we try our best to search all the potential eligible studies. Secondly, all the eligible studies are retrospective observational studies and most of them do not compare the baseline characteristics, CA characteristics, and initial neurological status before therapeutic hypothermia treatment. Thirdly, most of the included studies have not reported adjusted ORs. Finally, the eligible studies are conducted in only several regions including Korea, Japan, Singapore, and Australia. Thus, further prospective well-designed and multicentric random controlled studies with larger sample size should be conducted to validate our conclusions.

## Conclusions

We got a comprehensive result from the current meta-analysis that therapeutic hypothermia might be associated with favorable neurological outcomes and survival in adult patients undergoing ECPR after cardiac arrest. However, our result is a synthesis of retrospective observational studies, which are the low-level evidence, and should be treated carefully. Further prospective well-designed and multicentric randomized controlled trials with larger sample size are necessary before putting our result into clinical practices.

## Supplementary information

**Additional file 1: Table S1.** Modified Newcastle-Ottawa scale (mNOS) used in present study for cohort studies. **Table S3.** Modified Newcastle-Ottawa scale (mNOS) scores of our included studies for neurological outcomes. **Table S4.** Modified Newcastle-Ottawa scale (mNOS) scores of our included studies for patients’ survival. **Table S5.** Meta-regression analyses on characteristics of cardiac arrest for the associations of therapeutic hypothermia with favorable neurological outcomes in CA patients receiving ECPR.

**Additional file 2.** Detailed characteristics of the included studies.

## Data Availability

All data generated or analyzed during this study are included in the published article that listed in Table 1.

## Abbreviations

95%CI: 95% confidence interval
CA: Cardiac arrest
CCPR: Conventional cardiopulmonary resuscitation
CPC: Cerebral Performance Categories
ECLS: Extracorporeal life support
ECPR: Extracorporeal cardiopulmonary resuscitation
F: Fixed-effects model
IHCA: In-hospital cardiac arrest
LCI: Lower 95% confidence interval
mNOS: Modified Newcastle Ottawa Scale (mNOS)
OHCA: Out-of-hospital cardiac arrest
ORs: Odds ratios
P_begg_: *P*-value for Begg’s test
P_egger_: *P*-value for Egger’s test
P_hetero_: *P*-value for heterogeneity
R: Random-effects model
TTM: Targeted temperature management
UCI: Upper 95% confidence interval
