# Supplementary material for: Associations of therapeutic hypothermia with clinical outcomes in patients receiving ECPR after cardiac arrest: systematic review with meta-analysis
Source: Scand J Trauma Resusc Emerg Med. 2020 Jan 14;28:3. doi: 10.1186/s13049-019-0698-z (PMC6961259; doi:10.1186/s13049-019-0698-z)
Supplement: Supplementary file 1 — Additional file 1: Table S1. Modified Newcastle-Ottawa scale (mNOS) used in present study for cohort studies. Table S3. Modified Newcastle-Ottawa scale (mNOS) scores of our included studies for neurological outcomes. Table S4. Modified Newcastle-Ottawa scale (mNOS) scores of our included studies for patients’ survival. Table S5. Meta-regression analyses on characteristics of cardiac arrest for the associations of therapeutic hypothermia with favorable neurological outcomes in CA patients receiving ECPR. [file 13049_2019_698_MOESM1_ESM.docx]

Table S1. Modified Newcastle-Ottawa scale (mNOS) used in present study for cohort studies

| **1** | **Representativeness of exposed cohort (sampling bias)** |
| --- | --- |
|  | • Low Risk = Truly representative and Somewhat representative |
|  | • High Risk = Selected group or no description |
| **2** | **Selection of the non-exposed cohort** |
|  | • Low Risk = drawn from the same community as the exposed cohort |
|  | • High Risk =drawn from a different source or no description |
| **3** | **Demonstrate specific temperature in therapeutic hypothermia and control arms** |
|  | • Low Risk = demonstrate the specific temperature in |
|  | • High Risk = self-report or no description |
| **4** | **Comparability of cohorts on baseline characteristics** |
|  | • Low Risk = Patients' baseline characteristics between exposure and control arms were comparable including patients' age and gender, and etc.. |
|  | • High risk= Low Risk = Patients' baseline characteristics between exposure and control arms were not comparable including patients' age and gender, and etc., or unclear |
| **5** | **Comparability of cohorts on cardiac arrest characteristics** |
|  | • Low Risk = Patients' cardiac arrest characteristics between exposure and control arms were comparable including cardiac location, witnessed CA, bystander CPR attempt, rhythm, and etc.. |
|  | • High risk= Patients' cardiac arrest characteristics between exposure and control arms were comparable including cardiac location, witnessed CA, bystander CPR attempt, rhythm, and etc.. |
| **6** | **Comparability of cohorts on neurological status before intervention (therapeutic hypothermia)** |
|  | • Low Risk = Patients' initial neurological status before intervention was comparable |
|  | • High Risk = Patients' initial neurological status before intervention was not comparable or unclear |
| **7** | **Matching or adjusted analysis for associations of TH with prognosis (two stars, and the total stars of item 4, 5, and 7 should less than 2 or be equal 2)** |
|  | • Low Risk =Patients between exposure and control arms were matched or the confounding factors were adjusted by multivariate analysis |
|  | • High Risk =Patients between exposure and control arms were not matched or the confounding factors were not adjusted by multivariate analysis |
| **8** | **Assessment of outcome** |
|  | • Low Risk = secured records/primarily assessed by investigators or ICD codes |
|  | • High Risk = self-report or no description |
| **9** | **Was Follow up enough for outcome to occur** |
|  | • Low risk = assessment should be performed at hospital discharge or after one month |
|  | • High Risk = outcomes at hospital discharge of after one month were unknown |
| **10** | **Adequacy of follow up of cohort** |
|  | • Low risk= all patient included or <20% of patient lost randomly during follow up |
|  | • High Risk = >20% or completely not random or unacceptable exclusion |

The scores ranging from 0 to 9. When Item 7 was two stars, ignore Item 4 and 5.

Table S3. Modified Newcastle-Ottawa scale (mNOS) scores of our included studies for neurological outcomes

|  | **Maekawa et al, 2013** | **Kim et al, 2014** | **Kagawa et al, 2015** | **Choi et al, 2016** | **Dennis et al, 2017** | **Pang et al, 2017** | **Yukawa et al, 2017** | **Otani et al, 2018** | **Ryu et al, 2019** |
| --- | --- | --- | --- | --- | --- | --- | --- | --- | --- |
| Representativeness of exposed cohort (sampling bias) | 1 | 1 | 1 | 1 | 1 | 1 | 1 | 1 | 1 |
| Selection of the non-exposed cohort | 1 | 1 | 1 | 1 | 1 | 1 | 1 | 1 | 1 |
| Demonstrate specific temperature in TH and control arms | 1 | 1 | 1 | 1 | 1 | 1 | 1 | 1 | 1 |
| Comparability of cohorts on baseline characteristics | 1 | 0 | 1 | 0 | 0 | 1 | 0 | 0 | 0 |
| comparability of cohorts on cardiac arrest characteristics | 0 | 0 | 1 | 0 | 0 | 0 | 0 | 0 | 0 |
| Matching or adjusted analysis for associations of TH with prognosis | 0 | 0 | 0 | 0 | 0 | 2 | 0 | 2 | 2 |
| Comparability of cohorts on neurological status before intervention (therapeutic hypothermia) | 0 | 0 | 0 | 0 | 0 | 1 | 0 | 1 | 0 |
| Assessment of outcome - Neurological status | 1 | 1 | 1 | 1 | 1 | 1 | 1 | 1 | 1 |
| Was Follow up enough for outcome to occur – CPC 1-2 | 1 | 1 | 1 | 1 | 1 | 1 | 1 | 1 | 1 |
| Adequacy of follow up of cohort – CPC 1-2 | 1 | 1 | 1 | 1 | 1 | 1 | 1 | 1 | 1 |
| Total score | 7 | 6 | 8 | 6 | 6 | 9 | 6 | 9 | 8 |

Table S4. Modified Newcastle-Ottawa scale (mNOS) scores of our included studies for patients’ survival

|  | **Kagawa et al, 2012** | **Maekawa et al, 2013** | **Choi et al, 2016** | **Lee et al, 2016** | **Dennis et al, 2017** | **Pang et al, 2017** | **Goto et al, 2018** | **Han et al, 2019** | **Ryu et al, 2019** |
| --- | --- | --- | --- | --- | --- | --- | --- | --- | --- |
| Representativeness of exposed cohort (sampling bias) | 1 | 1 | 1 | 1 | 1 | 1 | 1 | 1 | 1 |
| Selection of the non-exposed cohort | 1 | 1 | 1 | 1 | 1 | 1 | 1 | 1 | 1 |
| Demonstrate specific temperature in TH and control arms | 1 | 1 | 1 | 1 | 1 | 1 | 1 | 1 | 1 |
| Comparability of cohorts on baseline characteristics | 0 | 1 | 0 | 0 | 0 | 1 | 0 | 0 | 0 |
| comparability of cohorts on cardiac arrest characteristics | 0 | 0 | 0 | 0 | 0 | 0 | 0 | 0 | 0 |
| Comparability of cohorts on neurological status before intervention (therapeutic hypothermia) | 0 | 0 | 0 | 0 | 0 | 1 | 1 | 0 | 0 |
| Matching or adjusted analysis for associations of TH with prognosis | 0 | 0 | 0 | 0 | 0 | 0 | 0 | 0 | 2 |
| Assessment of outcome - Mortality | 1 | 1 | 1 | 1 | 1 | 1 | 1 | 1 | 1 |
| Was Follow up enough for outcome to occur - Mortality | 1 | 1 | 1 | 1 | 1 | 1 | 1 | 1 | 1 |
| Adequacy of follow up of cohort - Mortality | 1 | 1 | 1 | 1 | 1 | 1 | 1 | 1 | 1 |
| Total score | 6 | 7 | 6 | 6 | 6 | 8 | 7 | 6 | 8 |

Table S5. Meta-regression analyses on characteristics of cardiac arrest for the associations of therapeutic hypothermia with favorable neurological outcomes in CA patients receiving ECPR

| **Characteristics** | **Coef.** | **SE** | **t** | **P-Value** | **95%CI** | |
| --- | --- | --- | --- | --- | --- | --- |
| IHCA proportion | -8.008 | 6.589 | -1.220 | 0.264 | -23.588 | 7.572 |
| Witnessed CA proportion | 29.126 | 28.898 | 1.010 | 0.347 | -39.207 | 97.460 |
| Bystander CPR attempt proportion | -16.092 | 17.102 | -0.940 | 0.383 | -57.938 | 25.754 |
| Shockable rhythm proportion | 2.977 | 23.106 | 0.130 | 0.901 | -51.659 | 57.614 |
| Cardiac origin CA proportion | 19.504 | 11.036 | 1.770 | 0.121 | -6.592 | 45.600 |
| ACS proportion | -6.220 | 20.678 | -0.300 | 0.774 | -56.817 | 44.376 |

Abbreviations: CA, cardiac arrest; IHCA, in of hospital cardiac arrest; 95%CI, 95% confidence interval; Coef., coefficient; SE, standard error; ACS,
